# Glutamate Activity Regulates and Dendritic Development of J-RGCs

**DOI:** 10.3389/fncel.2018.00249

**Published:** 2018-08-14

**Authors:** Eerik Elias, Ning Yang, Ping Wang, Ning Tian

**Affiliations:** ^1^Department of Ophthalmology and Visual Science, University of Utah School of Medicine, Salt Lake City, UT, United States; ^2^VA Salt Lake City Health Care System, Salt Lake City, UT, United States; ^3^Eye Center, Renmin Hospital of Wuhan University, Wuhan, China

**Keywords:** glutamate activity, dendritic morphology, development, retinal ganglion cell, activity-dependent plasticity, NMDAR, light deprivation

## Abstract

Retinal ganglion cells (RGCs) have a wide variety of dendritic architectures, which are critical for the formation of their function-specific synaptic circuitry. The developmental regulation of the dendrites of RGCs is thought to be subtype dependent. The purpose of this study is to characterize the dendritic development of a genetically identified RGC subtype, JamB RGCs (J-RGCs), and the roles of glutamate receptor activity on the dendritic development of these cells. We show that the dendrites of J-RGCs are strictly ramified in the outer portion of the inner plexiform layer (IPL) of the retina at the age of postnatal day 8 (P8), mimicking the ramification pattern of adults. However, several other important features of dendrites undergo substantial developmental refinement after P8. From P8 to P13, the dendritic development of J-RGCs is characterized by a dramatic increase of dendritic length and the size of the dendritic field. After eye opening, the dendritic development of J-RGCs is characterized by a tremendous decrease of the number of dendritic protrusions (spine-like structures) and a consolidation of the size of the dendritic field. To determine whether the dendritic development of J-RGCs is regulated by glutamatergic activity, we conditionally knocked out the expression of an obligatory subunit of N-methyl-D-aspartate receptors (NMDARs), NR1 (Grin1), in J-RGCs. We found that J-RGCs with the NMDAR mutation have decreased dendrite outgrowth and dendritic field expansion but increased number of dendritic protrusions before eye opening. To determine if visual experience regulates the development of J-RGC dendrites, we raised the mice in complete darkness after birth. Light deprivation prevented the decrease in the number of dendritic protrusions and the consolidation of the dendritic field of wild type (WT) mice after eye opening. However, light deprivation has no additional effect on the number of dendritic protrusions or the size of the dendritic field of J-RGCs with NMDAR mutation. Together, these results revealed the roles of light stimulation and NMDAR activity on the dendritic development of J-RGCs.

## Introduction

Neurons are one of the most morphologically diverse cell types due to the large variety of their dendritic patterns, which are specialized structures for synaptic formation (Whitford et al., 2002; Jan and Jan, 2010; Vaney et al., 2012). Retinal ganglion cells (RGCs) also have a wide variety of dendritic patterns, which are critical for specific circuit formation and function (Masland, 2001; Sun et al., 2002; Badea and Nathans, 2004; Coombs et al., 2007; Berson, 2008; Kim et al., 2008; Völgyi et al., 2009; Kay et al., 2011; Sanes and Masland, 2015). One well-known example is the JamB expressing RGCs (J-RGCs) in mouse retina, which orient their dendrites ventrally to form a polarized dendritic field. This polarized dendritic field is thought to be critical for J-RGCs to preferentially detect the directional movement in the visual field (Kim et al., 2008, 2010), have a color opponent receptive field (Joesch and Meister, 2016) and to have orientation selectivity of J-RGCs (Nath and Schwartz, 2017). In mammals, RGCs were initially classified into subtypes morphologically (Masland, 2001; Sun et al., 2002; Badea and Nathans, 2004; Coombs et al., 2007; Berson, 2008; Kim et al., 2008; Völgyi et al., 2009; Kay et al., 2011), then combined with functional features (Briggman and Euler, 2011; Briggman et al., 2011; Baden et al., 2013). Recently, RGCs are classified into at least 25 subtypes using a combination of morphological, functional and genetic features (Roska and Meister, 2014; Sanes and Masland, 2015). The formation of subtype specific dendritic architecture of RGCs is heterogeneous and dendrites of some subtypes of RGCs undergo dramatic structural refinement during postnatal development as they grow into the inner plexiform layer (IPL), stratify, and form synapses with bipolar and amacrine cells (Tian, 2008; Kim et al., 2010). Conflicting studies, however, disagree as to whether or not RGC dendritic development is activity dependent and increasing evidence suggests that the discrepancy of the activity dependency of the development of RGC dendrites might be the result of the cell subtype specific developmental processes (Bodnarenko and Chalupa, 1993; Tagawa et al., 1999; Guenther et al., 2004; Xu and Tian, 2007; Kerschensteiner et al., 2009; Chang et al., 2010). Therefore, characterizing the developmental profile of the dendrites of each subtype of RGCs and their activity-dependency will directly address this discrepancy.

Glutamate receptors, especially N-methyl-D-aspartate receptors (NMDARs), play important roles in the dendritic development and synaptic formation of the CNS (McAllister, 2000; Cline and Haas, 2008). Impairment of NMDAR function blocks the normal axonal/dendritic morphogenesis of optic tectal neurons, motor neurons and cortical neurons (Inglis et al., 1998; Rajan and Cline, 1998; Datwani et al., 2002; Hebbeler et al., 2002; Lee et al., 2005; Espinosa et al., 2009). NMDARs are expressed by all RGCs (Fletcher et al., 2000; Pourcho et al., 2001; Guenther et al., 2004; Zhang and Diamond, 2009) and NMDAR-mediated mechanisms have been postulated to regulate RGC axonal morphogenesis in zebrafish and *Xenopus* (Ben Fredj et al., 2010; Munz et al., 2014), dendritic morphogenesis and α-amino-3-hydroxy-5-methyl-4-isoxazolepropionic acid receptor (AMPAR) plasticity in RGCs of mice (Xu et al., 2010; Jones et al., 2012). In addition, light stimulation has been shown to regulate the development of RGC dendrites and their synaptic function (Fisher, 1979; Wingate and Thompson, 1994; Sernagor and Grzywacz, 1996; Tian and Copenhagen, 2001, 2003; Xu and Tian, 2007; Tian, 2008; Akimov and Rentería, 2014), as well as the expression and activity of NMDARs on RGCs (Fox et al., 1991; Carmignoto and Vicini, 1992; Xue and Cooper, 2001; Guenther et al., 2004). However, whether and how NMDARs regulate the dendritic morphogenesis of specific subtypes of RGCs has not been reported.

J-RGCs are a unique population of RGCs, which has a unique polarized dendritic architecture corresponding remarkably to their directional selective functionality (Kim et al., 2008, 2010), color opponent receptive field (Joesch and Meister, 2016) and orientation selectivity (Nath and Schwartz, 2017). It has been shown that the dendrites of J-RGCs undergo significant developmental refinement during early postnatal development (Kim et al., 2008, 2010). Therefore, we use this function-specific subtype RGCs as a model to investigate the roles of glutamate receptor-mediated activity in the dendritic development of RGCs. Our results showed that the dendrites of J-RGCs undergo an orderly developmental process during postnatal ages. The ramification of the dendrites of J-RGCs in IPL reaches the adult pattern before postnatal day 8 (P8), while other structural features of dendrites undergo substantial developmental refinement after P8. From P8 to P13, the dendritic development of J-RGCs is characterized by a dramatic increase in the dendritic length and the size of the dendritic field. After eye opening, the dendritic development of J-RGCs is characterized by a complete cease in dendritic growth, a decrease in the number of dendritic protrusions and a consolidation of the size of the dendritic field. Conditional mutation of NMDARs on J-RGCs resulted in decreased dendrite outgrowth and dendritic field expansion but an increased number of dendritic protrusions before eye opening. Light deprivation, on the other hand, prevented the developmental decrease in the number of dendritic protrusions and the consolidation of dendritic field of wild type (WT) mice after eye opening but with little effect on the dendritic structure of J-RGCs with cell-specific mutation of NMDARs. Together, these results have for the first time revealed the roles of light stimulation and NMDAR activity on the dendritic development of a genetically-identified functionally-specific subtype of RGCs.

## Materials and Methods

### Animals

JamB-CreER and Thy1-Stop-YFP (yellow fluorescent protein) mice were obtained from Dr. Joshua Sanes’ laboratory at Harvard University (Kim et al., 2008). These mice were generated on a C57BL/6 background and backcrossed with C57BL/6J mice 4–5 generations in our lab. Then the JamB-CreER mice were bred with the Thy1-Stop-YFP mice to create JamB-CreER:Thy1-Stop-YFP double transgenic mice and YFP is expressed specifically in J-RGCs upon intraperitoneal (IP) injection of Tamoxifen. These mice were used as “WT” controls for J-RGC dendritic morphology. B6.129S4-*Grin1*^tm2Stl^/J mice were obtained from the Jackson Laboratory (Bar Harbor, ME, USA). To generate these mice, the construct was electroporated into 129S4/SvJae derived J1 embryonic stem (ES) cells. Correctly targeted ES cells were injected into C57BL/6 blastocysts. The resulting chimeric animals were crossed to C57BL/6J mice. The colony was then backcrossed to C57BL/6J for eight generations prior to sending to The Jackson Laboratory Repository. Therefore, this mouse strain also has a C57BL background. JamB-CreER:Thy1-Stop-YFP:Grin1^flox/flox^ triple transgenic mice were generated by breeding JamB-CreER:Thy1-Stop-YFP mice with B6.129S4-*Grin1*^tm2Stl^/J mice. Therefore, both JamB-CreER:Thy1-Stop-YFP double transgenic mice and JamB-CreER:Thy1-Stop-YFP:Grin1^flox/flox^ triple transgenic mice are considered having C57BL/6 background. When the triple transgenic mice are treated with Tamoxifen, J-RGCs in the retina express YFP and stop the expressing of NR1 specifically in J-RGCs (Tsien et al., 1996). All mice were treated with IP injection of Tamoxifen (150 μg) at the ages of P1–3. The control animals were fed and housed under 12:12-h cyclic light/dark conditions. The average light intensity in the animal room during subjective day was 300–400 lux while the light intensity inside the cages during subjective day was 40 lux for control mice. Dark-reared animals were housed in conventional mouse cages, which were placed in a continuously ventilated light-tight box. The temperature and humidity inside the box were continuously monitored and controlled. All the procedures of daily monitoring and routine maintenance of dark-reared mice were conducted under infrared illumination. All animal procedures and care were preformed following protocols approved by the IACUC of the University of Utah in compliance with PHS guidelines and with those prescribed by the Association for Research in Vision and Ophthalmology (ARVO).

### Intraocular Injection

The procedure of intraocular injection of GluR antagonists and saline into mouse eyes has been described previously (Xu et al., 2010). Various glutamate receptor antagonists have been used for intraocular injection to pharmacologically manipulate the activity of glutamate receptors. Regardless the types of glutamate receptor analogous and concentration, the procedures of intraocular injection were the same. The actual concentrations of the injected chemicals were prepared so the volume of the solution injected was always 2 μl in order to have the solution evenly distributed inside the eye. For the injection procedure, the mice were anesthetized with Isoflurane (1%–5% Isoflurane mixed with room air delivered in a rate between 0.8–0.9 L/min) through a mouse gas anesthesia head holder (David KOPF Instruments, Tujunga, CA, USA) and local application of 0.5% proparacaine hydrochloride ophthalmic solution on each eye. Glass micropipettes made from borosilicate glass using a Brown-Flaming horizontal puller with very fine tip (about 10–15 μm diameter) were used for injection. The glass needle was mounted on a Nano-injection system, which could precisely control the amount of injected solution at the nl level. The glass needle was aimed to penetrate the eyeball near equator under a dissection microscope and a total of 2 μl solution was slowly injected into each eye. After the procedure, the mice were placed in a clean cage siting on a water blanket. The temperature of the water blanket was set at 33°C. Mice in this cage were continuously monitored until they completely recovered and then they were returned to their original cages. The procedures for anesthesia and intraocular injection fit the procedures described in the University of Utah IACUC Policies.

### Primary Antibodies

Rabbit polyclonal antibody against green fluorescent protein (GFP) conjugated with AlexaFluor 488 was purchased from Molecular Probes (Eugene, OR, USA; catalog No. A21311). This antibody was raised against GFP isolated directly from *Aequorea Victoria* and has been characterized by immunocytochemistry in granule cells (Overstreet-Wadiche et al., 2006), olfactory sensory neurons (Lèvai and Strotmann, 2003), and hippocampal neurons that express GFP (Huang et al., 2005). Antibody directed toward choline acetyltransferase (ChAT) was purchased from Millipore (Temecula, CA, USA; catalog No. AB144P). This polyclonal antibody was raised in goat against human placental enzyme and has been characterized by Western blotting, recognizing a band at 68–70 kD.

### Preparation of Retinal Whole-Mounts and Retina Sections for Fluorescent Imaging

J-RGC dendritic arbors were imaged on a whole mount retinal preparation while the dendritic ramification of J-RGCs in the IPL was imaged on a retinal slice preparation using confocal microscopy. The procedures for fluorescent immuno-labeling of YFP-expressing RGCs on retinal whole-mount and slide preparations have been described previously in detail (Tian and Copenhagen, 2003; Xu and Tian, 2007; Xu et al., 2010). In brief, mice were euthanized at P8, P13 or P30 with 100% CO_2_ followed by either cervical dislocation (for P30 mice) or decapitation (for P8 and P13 mice).

For retinal whole mount preparation, retinas were isolated and fixed in 4% paraformaldehyde (PFA) in 0.01 M phosphate-buffered saline (PBS; pH 7.4) for 30 min at room temperature. Fixed retinas were washed 10 min × 3 in 0.01 M PBS and incubated in blocking solution (10% normal donkey serum) at 4°C for 2 h. Next, retinas were incubated in rabbit polyclonal anti-GFP antibody conjugated with Alexa Fluor488 (1:500) for 7 days at 4°C. Retinas were then washed and flat mounted on Super-Frost slides (Fisher Scientific, Pittsburgh, PA, USA) with Vectashield mounting medium for fluorescence (Vector Laboratories, Burlingame, CA, USA).

For retina section preparation, the whole eyes were removed and fixed in 4% PFA for 2 h. Fixed eyes were washed three times for 10 min each in 0.01 M PBS and incubated in 15% sucrose for 2 h and then 30% sucrose at 4°C overnight. Eyes were than embedded in Tissue-Tek OCT compound (Sakura Finetek USA Inc., Torrance, CA, USA), sectioned vertically at 12–15 μm thickness on a Leica CM-3050-S cryostat microtome (Leica Biosystems, Wetzlar, Germany), and collected on Super-Frost Plus slides. A rabbit polyclonal anti-GFP antibody conjugated with Alexa Fluor488 (1:500) and a goat polyclonal anti-ChAT antibody (1:150) were used to label YFP-expressing J-RGCs and the dendritic plexus of cholinergic amacrine cells, respectively. A rhodamine red-conjugated donkey anti-goat (1:100) secondary antibody was used to reveal the anti-ChAT bindings. The secondary antibody was purchased from Jackson Immuno Research Laboratories (West Grove, PA, USA).

### Confocal Laser Scanning Microscopy and Quantitative Image Analysis

Fluorescent images were collected using a dual-channel Zeiss microscope (Carl Zeiss AG, Germany) with the C-Apochromat 40× 1.2 W Korr water immersion lens from retinal section or whole mount preparations. Multiple images of immunolabeled retinal sections were taken at steps of 0.4 μm. Several optical sections were assembled to achieve the final image. The brightness and contrast of the final images were adjusted in Adobe Photoshop CS5 (Adobe Systems Inc., San Jose, CA, USA). The dendritic stratification of each RGC was characterized by the ramification depth (peak dendritic location) and thickness (dendritic width) in the IPL, both of which were calculated by fitting the pixel intensities of the image of retinal cross section into Gaussian distribution using the software Igor (WaveMetrics, Lake Oswego, OR, USA). The IPL was defined as 0%–100% from the border of inner nuclear layer to the border of ganglion cell layer. This approach has been described in details previously (Xu and Tian, 2007). Image stacks of YFP-expressing RGCs in whole-mount retinas were collected at intervals of 0.5 μm. IPLab software (Scanalytics, Inc., Fairfax, VA, USA) was used to align multistacks of images together. The entire dendritic tree of each RGC was traced in the 3D stack using Neurolucida software (Neurolucida 2000, Microbrightfield, Williston, VT, USA). The total dendritic length, the number of dendritic branches, and the number of filopodia were measured by the software based on the tracing using the approach we previously described (Xu et al., 2010). For the measurement of the size of the dendritic field, the image stack of each RGC was maximum projected into a single frame and then the dendritic field was outlined by connecting the tip of terminal dendrites to form a convex enclosure using Neurolucida 2000. The size of the dendritic field was calculated by the software based on the convex enclosure. Because the length of dendrites and the size of the dendritic field of RGCs vary with the eccentricity of the cells in the retina, we only included the RGCs with their soma located ±500 μm around the equator of the eye ball (Supplementary Figure S1).

### Statistical Analysis

Data are all presented as mean ± SEM in the text and figures. ANOVA, *post hoc* (Bonferroni-Dunn) tests were used to determine the significance of the difference between more than two means. Student *t*-tests were used to examine the difference between two means. All of the statistical tests were performed with STATVIEW (Abacus Concepts, Berkeley, CA, USA).

## Results

### The Development of J-RGC Dendrites Undergo an Orderly Process During Postnatal Ages

**Figure 1:**
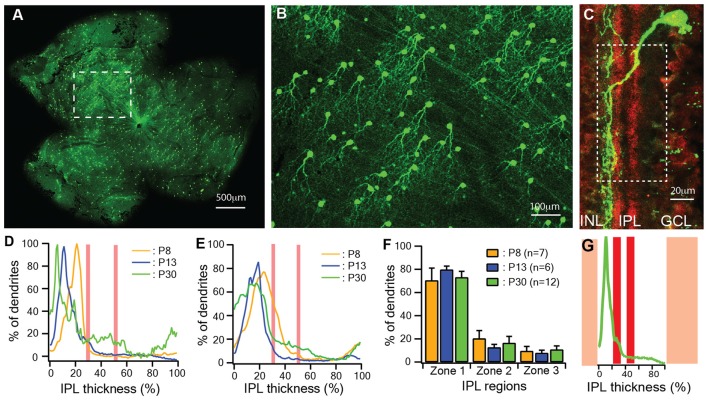
The dendritic ramification of JamB retinal ganglion cells (J-RGCs) reaches adult pattern early during postnatal development. The retinas of JamB-CreER:Thy1-YFP mice were stained with an anti-green fluorescent protein (GFP) antibody and flat mounted for dendritic tree imaging or sectioned for ramification analysis. **(A)** A representative image of a whole mount retina of a JamB-CreER:Thy1-YFP mouse. **(B)** An enlarged view of the retina area indicated by the dashed line box in **(A)** showing YFP expressing J-RGCs. **(C)** Immunolabeling of cholinergic amacrine cell dendritic plexus (red) and the dendritic plexus of a J-RGC (green) of a cross section of a P30 JamBCreER:Thy1-YFP mouse retina. **(D)** Representative dendritic density distribution curves of three J-RGCs from a postnatal day 8 (P8), a P13 and a P30 retina. **(E)** Average dendritic density profile as a function of the inner plexiform layer (IPL) depth measured as the fluorescent intensity of the J-RGCs at three different ages (P8: 2 mice, 7 cells; P13: 2 mice, 6 cells; P30: 3 mice, 12 cells). **(F)** Average dendritic distribution of the J-RGCs in three IPL zones at three ages. **(G)** The fluorescent intensity profile of the dendritic plexus of the J-RGC in **(C)** plotted as a function of the IPL depth (green). The red bands indicate the cholinergic amacrine cell dendritic plexus. The yellow bands indicate the GCL and INL.

It has been shown that the dendrites of J-RGCs undergo developmental refinement early in postnatal ages (Kim et al., 2008, 2010). We first quantitatively characterized the developmental profile of the dendritic ramification of J-RGCs at three critical ages: immediately before RGCs receive glutamate synaptic inputs (P8; Bansal et al., 2000; Johnson et al., 2003), around the time of eye opening (P13) and in young adult (P30). The J-RGCs in the JamB-CreER:Thy1-YFP double transgenic mice show strong YFP expression as early as P5 (Kim et al., 2008, 2010), which reveals all details of their dendrites (Figures 1A,B). To assess the J-RGC dendritic distribution in the IPL precisely, we measured the pixel intensities of YFP signals of dendrites of individual J-RGCs (Figure 1C). The average pixel intensity of a region of interest (white dash line box in Figure 1C) was calculated from a z-stack of the confocal image and plotted as a function of the IPL thickness for the estimation of a normalized dendritic density distribution (Figure 1G; Xu and Tian, 2007). The IPL was defined as 0%–100% from the border of inner nuclear layer to the border of ganglion cell layer and divided into three regions based on the position of cholinergic amacrine cell dendritic plexus in the IPL. Figure 1D shows representative dendritic density distribution curves of three J-RGCs from a P8, a P13 and a P30 retina. Although the dendritic density of these three J-RGCs peak at different locations in the outer portion of the IPL, the average dendritic density distributions of J-RGCs at the three ages substantially overlapped with each other (Figure 1E). Quantitatively, the average dendritic distributions of J-RGCs from the three ages in the IPL are not different statistically (Figure 1F). These results are consistent with the previous report that the dendrites of J-RGCs reach the adult ramification pattern in the IPL early during postnatal development (Kim et al., 2010).

**Figure 2:**
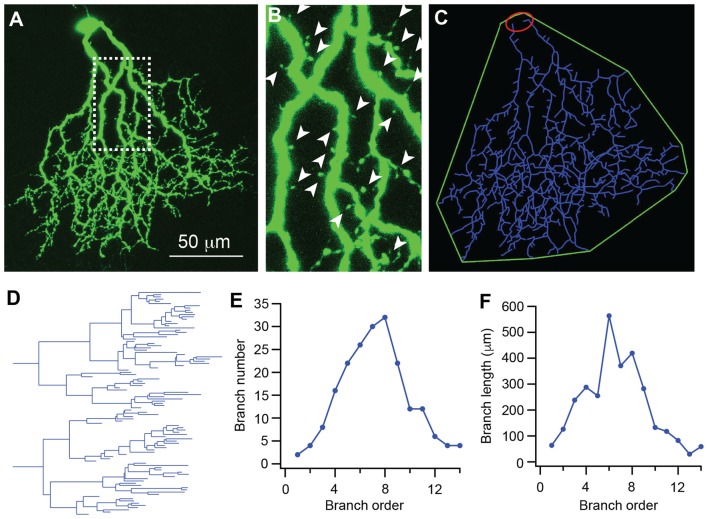
The dendritic pattern of J-RGCs is quantitatively analyzed. The dendritic pattern of J-RGCs was quantitatively analyzed. **(A)** Maximum projection of the confocal image of a J-RGC. **(B)** An enlarged view of the dendrites indicated by the dashed line box of **(A)** showing the dendritic protrusion (arrowheads). **(C)** The tracing result of the J-RGC showing the soma (red), the dendrites (blue), and the dendritic field (green). **(D)** The dendrogram of the tracing result of the J-RGC shown in **(C)**. The total length of dendrites, the number of dendritic branches, and the number of dendritic protrusions were quantified based on this dendrogram. **(E)** The plot of the number of dendritic branches as a function of branch order of the cell in **(A)**. **(F)** The plot of dendritic branch length as a function of branch order of the same cell in **(A)**.

We then quantitatively analyzed several important dendritic features of J-RGCs during postnatal development. To estimate the dynamics of dendritic growth and branching during postnatal development (Xu et al., 2010; Yang et al., 2010), we quantified the age-dependent changes in the total number of the dendritic protrusions of J-RGCs (Figure 2B; Munz et al., 2014), total length of the dendrites (Figures 2A,C,D), number of dendritic branches (Figure 2E), length of dendritic branches (Figure 2F), and size of the dendritic field (Figure 2C) of each J-RGC.

**Figure 3:**
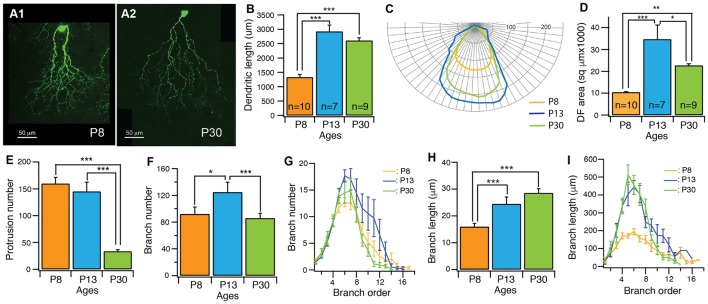
The dendritic pattern of J-RGCs is developmentally refined. The dendritic patterns of J-RGCs were quantitatively analyzed from three groups of JamB-CreER:Thy1-YFP mice at the ages of P8 (3 mice, 10 cells), P13 (2 mice, 7 cells) and P30 (3 mice, 9 cells), respectively. **(A)** Maximum projections of confocal images of J-RGCs from a P8 mouse **(A1)** and a P30 mouse **(A2)**. **(B)** The average total length of dendrites of J-RGCs of the three age groups. **(C)** The average polar plots of the dendritic field (DF) of the J-RGCs of three age groups shown in **(B)**. These average polar plots were calculated by aligning the polar plots of individual J-RGCs based on the soma and the peak of the polar plots. **(D)** The average size of DF of J-RGCs of three age groups shown in **(B)**. **(E)** The average number of dendritic protrusions of J-RGCs of the same three age groups. **(F)** The average number of dendritic branches of the three groups of J-RGCs. **(G)** The plot of the average number of dendritic branch as a function of branch order of the three groups of J-RGCs. **(H)** The average length of dendritic branches of J-RGCs of the three groups. **(I)** The plot of dendritic branch length as a function of branch order of the three groups of J-RGCs. *P < 0.05; **P < 0.01; and ***P < 0.001.

Our results showed that the dendrites of J-RGCs undergo significant developmental refinement (Figure 3A). Before eye opening (P13), the developmental refinement of dendrites was characterized by active dendritic elongation, a large number of dendritic protrusions and rapid expansion of the dendritic field. From P8 to P13, the total length of dendrites increased by 2.2-fold (from 1332.2 μm ± 97.8 μm at P8–2919.6 μm ± 229.4 μm at P13, *p* < 0.0001; Figure 3B), the size of the dendritic field increased by 3.3-fold (from 10391.7 μm^2^ ± 267.4 μm^2^ at P8–34733.7 μm^2^ ± 6489.8 μm^2^ at P13, *p* < 0.0001; Figures 3C,D), and the number of dendritic protrusions remained at a high level during this period (159.7 ± 11.7 and 145 ± 17.4 for P8 and P13 J-RGCs, respectively; Figure 3E). Additional analysis of the dendritic structure of J-RGCs at P8 and P13 showed that the number of dendritic branches increased by 1.4-fold (from 92.3 ± 10.5 at P8 to 124.7 ± 15 at P13, *p* = 0.05; Figure 3F), while the length of dendritic branches increased by 1.9-fold (from 116.4 μm ± 7.6 μm at P8 to 219.7 μm ± 15.7 μm at P13, *p* < 0.0001; Figure 3G). Therefore, the total dendritic elongation and dendritic field expansion is due to both the elongation of individual dendritic branches and addition of new dendritic branches. Interestingly, the extent of dendritic elongation was most significant for the dendritic branch orders 4–8 (Figure 3I) without a proportional increase in the number of dendritic branches in this range (Figure 3G), indicating that the developmental changes of the dendritic length and the size of the dendritic field of J-RGCs is unlikely to be the result of developmental retina expansion.

After eye opening, the developmental refinement of J-RGC dendrites was characterized by significant reduction of the number of dendritic protrusions (Figure 3E), a complete stop of the dendritic elongation (Figure 3B), a significant consolidation of the size of the dendritic field (Figures 3C,D), and the pruning of terminal dendritic branches (Figure 3F). Specifically, the number of dendritic protrusions decreased by 77% (from 145 ± 17.4 at P13 to 33.3 ± 2.9 at P30, *p* < 0.0001) and the size of the dendritic field decreased by 35% (from 34733.7 μm^2^ ± 6489.8 μm^2^ at P13 to 22712.3 μm^2^ ± 772.3 μm^2^ at P30, *p* = 0.0133). However, the total length of dendrites remained at similar levels (2919.6 μm ± 229.4 μm vs. 2607.8 μm ± 94.9 μm for P13 and P30 J-RGCs, respectively; *p* = 0.1376). Further analysis showed that the dendritic field consolidation is associated with a significant decrease in the number of dendritic branches from 124.7 ± 15 at P13 to 85.9 ± 7.5 at P30 (*p* = 0.024; Figure 3F), mostly in the higher order of dendritic branches (Figure 3G), but very little change in the total length of dendrites (Figure 3B) and the average length of dendritic branches was observed (24.5 μm ± 2.5 μm vs. 28.5 μm ± 1.6 μm for P13 and P30 J-RGCs, respectively, *p* = 0.2024; Figures 3H,I).

Taken together, the dendrites of J-RGCs undergo an orderly developmental refinement. The dendritic ramification depth of J-RGCs in the IPL reaches the adult level before P8. Then the dendritic growth, branching and dendritic field expansion are completed around the time of eye opening. Finally, the development of dendrites ends by pruning of terminal dendrites and dendritic protrusions, which is associated with a consolidation of the size of the dendritic field before P30. Because the developmental refinement of J-RGC dendrites occurred during the time period in which RGCs start to receive spontaneous glutamatergic synaptic inputs before eye opening (Bansal et al., 2000; Johnson et al., 2003) and light evoked glutamatergic synaptic inputs after eye opening, it opens the possibility that developmental refinement of J-RGC dendrites may be driven by glutamatergic synaptic activity.

### Glutamate Receptor Activity Is Required for the Development of J-RGC Dendrites

**Figure 4:**
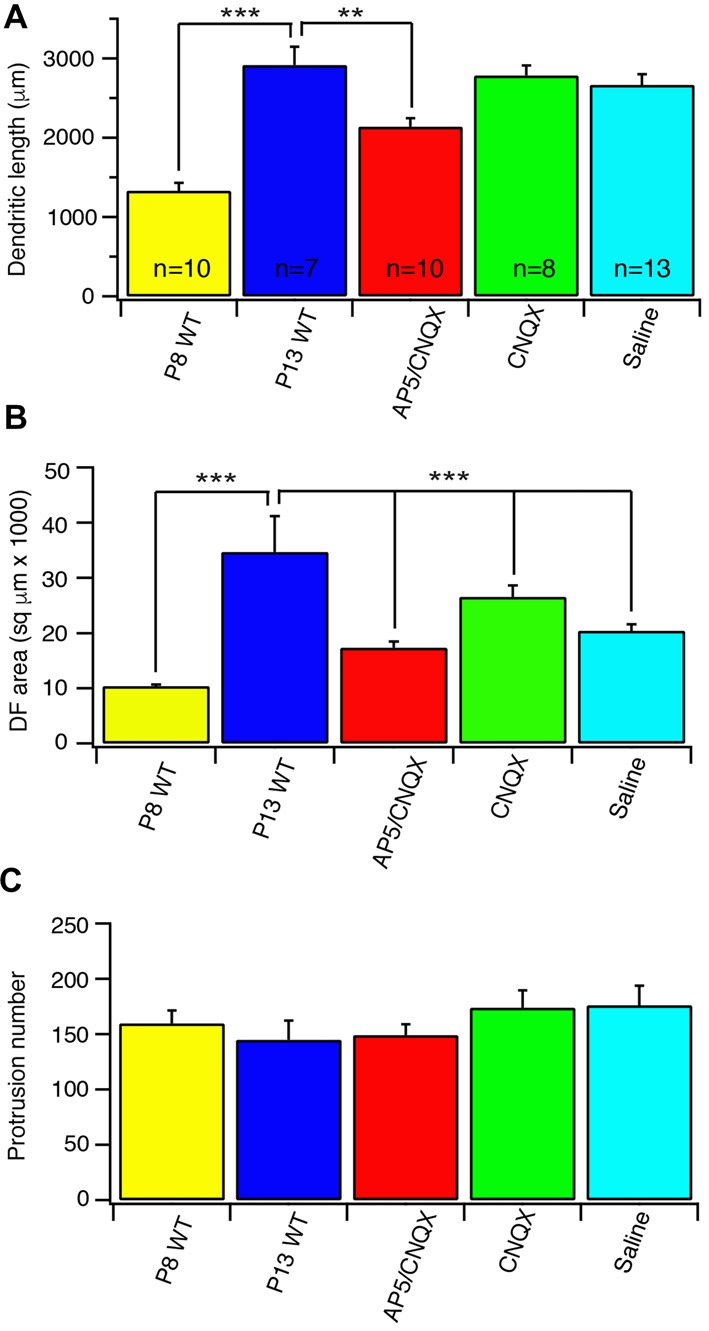
Blockage of glutamate receptor activity retarded the developmental refinement of J-RGC dendrites. The dendritic pattern of J-RGCs was quantitatively analyzed at the age of P13 of JamB-CreER:Thy1-YFP mice with intraocular injection of CNQX or PA5+CNQX for 4 days from P9 to P12. The results were compared with that of JamB-CreER:Thy1-YFP mice at the ages of P8 (P8 wild type (WT): 3 mice, 10 cells) and P13 (P13 WT: 2 mice, 7 cells) without injection and the mice with 4 days intraocular injection of saline. **(A)** The average total dendritic length of J-RGCs at ages of P8 (P8 WT), P13 (P13 WT), P13 mice treated with intraocular injection of CNQX (CNQX: 3 mice, 8 cells), CNQX+AP5 (AP5/CNQX: 3 mice, 10 cells) or saline (Saline: 3 mice, 13 cells). **(B)** The average sizes of DF of J-RGCs of the same five groups of mice as shown in **(A)**. **(C)** The average number of dendritic protrusions of J-RGCs of the same five groups of mice as shown in **(A)**. *P < 0.05; **P < 0.01; and ***P < 0.001.

Glutamate receptors, especially NMDARs, have been found to play important roles in the development of CNS neurons (Inglis et al., 1998; Rajan and Cline, 1998; Cline and Haas, 2008; McAllister, 2000; Datwani et al., 2002; Hebbeler et al., 2002; Lee et al., 2005; Espinosa et al., 2009) as well as the axonal and dendritic morphogenesis of RGCs (Ben Fredj et al., 2010; Xu et al., 2010; Jones et al., 2012; Munz et al., 2014). To test the possibility that the developmental refinements of J-RGC dendrites during postnatal age is regulated by glutamatergic activity, we blocked glutamatergic synaptic activity with intraocular injections of either the antagonist of AMPARs, CNQX, alone or CNQX with a NMDAR antagonist, AP5, from P8 to P13. Examination of the dendrites of J-RGCs showed that intraocular injection of CNQX+AP5 reduced the developmental elongation of J-RGC dendrites by 27% (2919.6 μm ± 229.4 μm vs. 2139.4 μm ± 103.5 μm for control and CNQX+AP5 treated J-RGCs, *p* = 0.01) while CNQX alone or saline had no significant effect on J-RGC dendritic length (*p* = 0.62 and 0.47, respectively), suggesting that NMDAR might play an important role in dendritic growth of J-RGCs (Figure 4A). In addition, the developmental expansion of dendritic field was reduced by 50% in eyes with injection of CNQX+AP5 (34733.7 μm^2^ ± 6489.8 μm^2^ vs. 17332.1 μm^2^ ± 1115.6 μm^2^ for control and CNQX+AP5 treated J-RGCs, *p* = 0.03, Figure 4B). However, CNQX alone or saline injection also reduced size of the dendritic field by 27% and 43% (34733.7 μm^2^ ± 6489.8 μm^2^ for control, 25282.6 μm^2^ ± 2243.6 μm^2^ for CNQX treated J-RGCs, and 19925 μm^2^ ± 911.7 μm^2^ for saline treated J-RGCs, *p* = 0.2 and 0.06, respectively), suggesting that the procedure of intraocular injection *per se* might have retarded the developmental expansion of the size of the dendritic field of J-RGCs (Figure 4B).

To further determine whether NMDARs play critical roles in the developmental refinements of J-RGC dendrites, we generated a triple transgenic mouse line (JamB-CreER:Thy1-YFP:Grin1^flox/flox^), in which the expression of the obligatory subunit of NMDAR, NR1, in J-RGCs is selectively blocked upon the activation of the CreER (Tsien et al., 1996), while the expression of YFP in J-RGCs is selectively activated in the mutant J-RGCs. We activated the NR1 knockout and YFP expression in J-RGCs at the ages of P1–3 and examined the dendrites of J-RGCs at the ages of P13 and P30, respectively.

**Figure 5:**
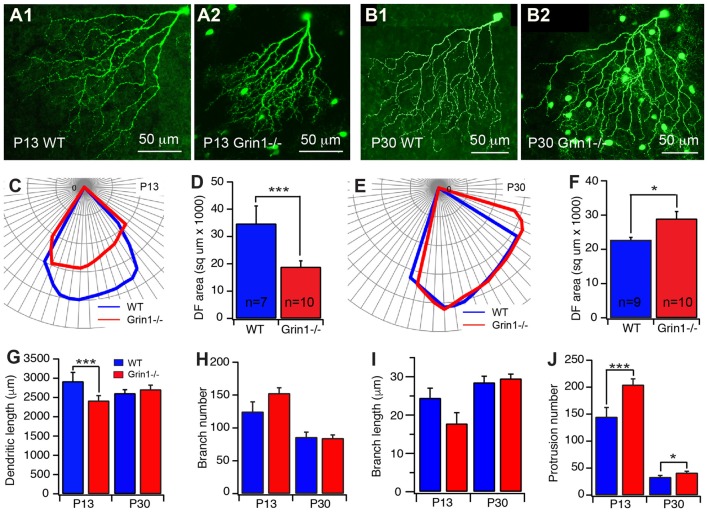
Cell type specific mutation of N-methyl-D-aspartate receptor (NMDAR) retarded the developmental refinement of J-RGC dendrites. The dendritic patterns of J-RGCs were quantitatively analyzed at the ages of P13 and P30 of JamB-CreER:Thy1-YFP mice (WT) and JamB-CreER:Thy1-YFP:Grin1−/− mice (Grin1−/−). **(A)** Representative maximum projections of confocal images of J-RGCs from a P13 JamB-CreER:Thy1-YFP mouse **(A1)** and a P13 JamB-CreER:Thy1-YFP:Grin1−/− mouse **(A2)**. **(B)** Representative maximum projections of confocal images of J-RGCs from a P30 JamB-CreER:Thy1-YFP mouse **(B1)** and a P30 JamB-CreER:Thy1-YFP:Grin1−/− mouse **(B2)**. **(C)** The polar plots of the DFs of the J-RGCs shown in (**A1**; blue) and (**A2**; red). **(D)** The average sizes of DFs of J-RGCs of JamB-CreER:Thy1-YFP mice (2 mice, 7 cells) and JamB-CreER:Thy1-YFP:Grin1−/− mice (3 mice, 10 cells) at P13. **(E)** The polar plots of the DFs of the J-RGCs shown in panels B1 (blue) and B2 (red). **(F)** The average sizes of DFs of J-RGCs of JamB-CreER:Thy1-YFP mice (3 mice, 9 cells) and JamB-CreER:Thy1-YFP:Grin1−/− mice (3 mice, 10 cells) at P30. **(G)** The average total length of dendrites of J-RGCs of JamB-CreER:Thy1-YFP mice and JamB-CreER:Thy1-YFP:Grin1−/− mice at P13 and P30. **(H)** The average number of dendritic branches of J-RGCs of JamB-CreER:Thy1-YFP mice and JamB-CreER:Thy1-YFP:Grin1−/− mice at P13 and P30. **(I)** The average length of dendritic branches of J-RGCs of JamB-CreER:Thy1-YFP mice and JamB-CreER:Thy1-YFP:Grin1−/− mice at P13 and P30. **(J)** The average number of dendritic protrusions of J-RGCs of JamB-CreER:Thy1-YFP mice and JamB-CreER:Thy1-YFP:Grin1−/− mice at P13 and P30. *P < 0.05; **P < 0.01; and ***P < 0.001.

Our results showed that selective knocking out of NR1 on J-RGCs reduces the expansion of the size of the dendritic field, the dendritic elongation and the number of dendritic protrusions before eye opening (Figure 5A). Quantitatively, the size of the dendritic field of Grin1^flox/flox^ J-RGCs at P13 was reduced by 46% in comparison with the age-matched controls (34733.7 μm^2^ ± 6489.83 μm^2^ vs. 18875.2 μm^2^ ± 2229.08 μm^2^, *p* = 0.0002; Figures 5C,D). The reduction of the size of the dendritic field is associated with a 17% shorter in the total dendritic length of Grin1^flox/flox^ J-RGCs (2417.3 μm ± 130.5 μm vs. 2919.6 μm ± 229.4 μm for Grin1^flox/flox^ and Grin1+/+ J-RGCs, respectively; *p* = 0.0158; Figure 5G) and a 41% increase of the dendritic protrusions (204.6 ± 10.8 vs. 145 ± 17.4 for Grin1^flox/flox^ and Grin1+/+ J-RGCs, respectively, *p* = 0.0046; Figure 5J). These results are similar to those seen following the intraocular injection of CNQX+AP5 (Figure 4) and our previous study (Xu et al., 2010).

However, the size of the dendritic field of Grin1^flox/flox^ J-RGCs at P30 was not smaller but 27% bigger than that of age-matched controls (28953.9 μm^2^ ± 2088.1 μm^2^ vs. 22712.3 μm^2^ ± 772.3 μm^2^, *p* = 0.0167; Figures 5B,E,F) accompanied by a slight increase in the number of dendritic protrusions (24%, Figure 5J) without a significant difference in the total dendritic length (Figure 5G). On the other hand, the defects in the size of the dendritic field and dendritic elongation of the Grin1^flox/flox^ J-RGCs are not associated with a significant defect of dendritic branching (Figures 5H,I). Together these results show that NMDARs are required for the development of J-RGC dendrites, especially before eye opening.

### Potential Interplay of Visual Stimulation and NMDAR Activity on the Development of J-RGC Dendrites

**Figure 6:**
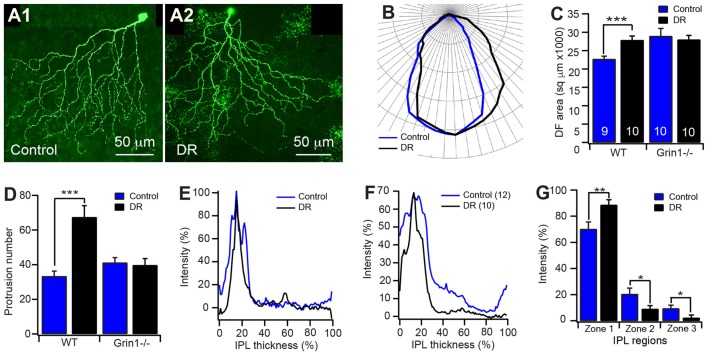
Potential interplay of visual stimulation and NMDAR activity on the development of J-RGC dendrites. The dendritic pattern of J-RGCs of JamB-CreER:Thy1-YFP:Grin1−/− (Grin1−/−) and JamB-CreER:Thy1-YFP (WT) mice raised in constant darkness from birth to P30 was quantitatively analyzed. The results were compared with that of age and genetic background matched mice raised under cyclic light/dark conditions. **(A)** Representative maximum projections of confocal images of J-RGCs of P30 JamB-CreER:Thy1-YFP mice raised under cyclic light/dark conditions **(A1)** and constant darkness from birth **(A2)**. **(B)** The average polar plots of the DFs of the J-RGCs of JamB-CreER:Thy1-YFP mice (3 mice, 9 cells) raised under cyclic light/dark conditions (blue) and constant darkness (black, 3 mice, 10 cells). **(C)** The average sizes of DFs of J-RGCs of JamB-CreER:Thy1-YFP (WT) and JamB-CreER:Thy1-YFP:Grin1−/− (Grin1−/−, 3 mice, 10 cells for both control and DR) mice raised under cyclic light/dark conditions (control) and in constant darkness (DR). **(D)** The average number of dendritic protrusions of J-RGCs of JamB-CreER:Thy1-YFP and JamB-CreER:Thy1-YFP:Grin1−/− mice raised under cyclic light/dark conditions and in constant darkness. **(E)** Representative dendritic density distribution curves as a function of the IPL depth of J-RGCs of JamB-CreER:Thy1-YFP mice raised under cyclic light/dark conditions and in constant darkness. **(F)** Average dendritic density profile as a function of the IPL depth of J-RGCs of JamB-CreER:Thy1-YFP mice raised under cyclic light/dark conditions and in constant darkness. **(G)** The average dendritic density distribution of the J-RGCs in three IPL zones of JamB-CreER:Thy1-YFP mice raised under cyclic light/dark conditions and in constant darkness. *P < 0.05; **P < 0.01; and ***P < 0.001.

Light stimulation has been shown to regulate the dendritic development of RGCs (Fisher, 1979; Wingate and Thompson, 1994; Sernagor and Grzywacz, 1996; Tian and Copenhagen, 2001, 2003; Xu and Tian, 2007; Tian, 2008; Akimov and Rentería, 2014) as well as the expression and activity of NMDARs on RGCs (Fox et al., 1991; Carmignoto and Vicini, 1992; Xue and Cooper, 2001; Guenther et al., 2004). We then investigated the potential interplay between light stimulation and the activity of NMDARs on the development of the dendrites of J-RGCs. We reared JamB-CreER:Thy1-YFP:Grin1^flox/flox^ and JamB-CreER:Thy1-YFP:Grin1+/+ (WT) mice in constant darkness from birth to P30 and examined the dendrites of J-RGCs in these two strains of mice. Our results showed that dark rearing increases the sizes of the dendritic field of Grin1+/+ J-RGCs by 23% in comparison with their age- and genetic-matched controls (22712.3 μm^2^ ± 772.3 μm^2^ vs. 27852 μm^2^ ± 1122.3 μm^2^ for mice raised under cyclic light/dark conditions and constant darkness, *p* = 0.0017; Figures 6A–C) but has no effect on the sizes of the dendritic fields of JamB-CreER:Thy1-YFP:Grin1^flox/flox^ mice (28953.9 μm^2^ ± 2088.1 μm^2^ vs. 33055.9 μm^2^ ± 2450.4 μm^2^ for mice raised under cyclic light/dark conditions and constant darkness, *p* = 0.2192; Figure 6C). Therefore, mutation of NMDARs alone increases the sizes of the dendritic fields to a similar size of dark reared Grin1+/+ J-RGCs while dark rearing of JamB-CreER:Thy1-YFP:Grin1^flox/flox^ mice has no additional effect.

In addition, the number of dendritic protrusions has been considered an indication of the activity of dendritic growth and synaptic formation. Additionally, neurons with less guided dendritic growth tends to have more dendritic protrusions (Munz et al., 2014). Consistently, the J-RGCs have large numbers of dendritic protrusions before eye opening (159.7 ± 11.7 and 145 ± 17.4 at P8 and P13, respectively) and the numbers of dendritic protrusions were reduced by 77% after eye opening (33.3 ± 2.9 at P30; Figure 3E). Dark reared JamB-CreER:Thy1-YFP:Grin1+/+ mice limited the developmental decrease in the number of dendritic protrusions. Therefore, the number of dendritic protrusions of dark reared Grin1+/+ J-RGCs is 2-fold higher than that of age-match controls raised under cyclic light conditions (33.3 ± 2.9 and 67.4 ± 6.6 for cyclic light and dark reared J-RGCs, respectively; *p* < 0.0001; Figure 6D). However, dark rearing has no additional effect on the number of dendritic protrusions of Grin1^flox/flox^ J-RGCs (Figure 6D). Taken together with the observation that mutation of NMDAR on J-RGCs increased the number of dendritic protrusions at P13, these results imply that NMDARs might play different roles in the synaptic formation of J-RGCs before and after eye opening.

Furthermore, light deprivation had no significant effect on the dendritic length and the number of dendritic branches of both Grin1+/+ and Grin1^flox/flox^ J-RGCs (data not shown). For the dendritic ramification in the IPL, J-RGCs of dark reared mice seem to ramify more dendrites in the outer IPL and reduced the density of dendrites in the inner IPL (Figures 6E–G). Overall, light deprivation seems to have specific effects on the developmental refinement of the size of the dendritic field and the number of dendritic protrusions. However, the effects of dark rearing on both of these two structural features are absent in J-RGCs with mutation of NMDARs.

## Discussion

In this study, we characterized the developmental profile of the dendrites of a genetically identified subtype of RGC, J-RGCs, and determined the roles of NMDARs and visual experience on the dendritic development of J-RGCs. Our results showed that the dendrites of J-RGCs undergo an orderly developmental process during postnatal ages. The dendritic ramification of J-RGCs in IPL reaches the adult pattern early during postnatal development, while other structural features of dendrites undergo substantial developmental refinement before and after eye opening. Before eye opening, the dendritic development of J-RGCs was characterized by a dramatic increase of dendritic length, the number of dendritic branches and the size of the dendritic field. Following eye opening, the dendritic refinement was characterized by a decrease in the number of dendritic protrusions and a consolidation of the size of the dendritic field. J-RGC specific mutation of NMDARs resulted in decreased dendrite growth, retarded dendritic field expansion and an increased number of dendritic protrusions before eye opening. Light deprivation of WT mice prevented the developmental decrease of the number of dendritic protrusions and the consolidation of dendritic field after eye opening. However, dark rearing of mice with J-RGC specific NMDAR mutation from birth has no effect in the number of dendritic protrusions and the size of the dendritic field. Taken together, these results at the first time revealed the roles of visual experience and NMDAR-mediated activity on the dendritic development of a specific subtype of RGCs.

### RGC Dendritic Morphogenesis

Dendrites of neurons are specialized structures to receive and process synaptic inputs from other neurons and thus provide anatomic basis for their specific functions. The size and shape of dendritic arbors of neurons affect the number and type of synaptic inputs, as well as the wiring and function of the synaptic circuits (Häusser et al., 2000; Parrish et al., 2007; Branco et al., 2010; Branco and Häusser, 2011; Gidon and Segev, 2012; Lavzin et al., 2012). J-RGCs are a unique population of RGCs in which dendritic architecture corresponds remarkably to their function (Kim et al., 2008, 2010). In adults, J-RGCs have a unique polarized dendritic field, which aligns in a dorsal-to-ventral direction across the retina. This polarized dendritic field is thought to be the determinative factor for their direction selective response to upward moving targets in the visual field (Kim et al., 2008, 2010), color opponent center-surround receptive field (Joesch and Meister, 2016) and orientation selectivity (Nath and Schwartz, 2017). However, how this uniquely polarized dendritic field is formed during development has not been fully characterized.

Dendrite morphogenesis is a complex but well-orchestrated process. Although individual neurons have unique developmental patterns of their dendrites, the critical steps in dendritic morphogenesis are similar for most CNS neurons. First, neurons must grow dendrites to form the dendritic field. In this stage, dendritic branches initially appear as filopodia-like dendritic protrusions and then morph into growth cone-like structures and extend to stable branches (Dailey and Smith, 1996). After the dendritic field reaches defined borders, the dendritic growth is restrained and the dendritic arbors are modified via pruning of excessive and inappropriate dendritic branches (Wässle et al., 1981; Gao et al., 1999; Puram and Bonni, 2013).

Developmentally, J-RGCs undergo significant refinement of their dendritic ramification in the IPL postnatally. J-RGCs ramify their dendrites throughout the entire IPL before P5 and then gradually eliminate their dendrites in the inner IPL and expand their dendrites in the outer portion of the IPL. By P12, they acquire adult pattern of dendritic ramification in the IPL (Kim et al., 2010). Our results confirmed this developmental refinement of dendritic ramification of J-RGCs in the IPL. However, other structural features of the dendrites of J-RGCs undergo prolonged developmental refinement similar to the common theme of dendritic morphogenesis of CNS neurons (Wässle et al., 1981; Dailey and Smith, 1996; Gao et al., 1999; Puram and Bonni, 2013). The early dendritic elongation, branching and dendritic field expansion occur before eye opening. Consistent with the high dynamic dendritic activity, many filopodia-like dendritic protrusions studded on the dendrites of J-RGCs during this period. After eye opening, J-RGCs completely cease the dendritic growth and branching. In addition, J-RGCs modify their dendritic field by pruning a significant number of terminal dendrites. With the increased visual activity, the number of dendritic protrusions is dramatically reduced after eye opening, consistent with the final step of the common scheme of dendritic morphogenesis (Orner et al., 2014; Puram and Bonni, 2013).

### NMDARs and RGC Dendritic Morphogenesis

It is well documented that spontaneous synaptic activity before eye opening is critical for the normal development of RGC dendrites. In early developing vertebrate retinas, RGCs fire periodic bursts of action potentials that are highly correlated and propagate across the RGC layer in a wave-like fashion (Wong, 1999). These spontaneous retina waves are mainly mediated by excitatory neurotransmission with a developmental shift from cholinergic to glutamatergic synaptic transmission (Wong, 1999; Wong et al., 2000). Within the first postnatal week of mice, retinal waves are only mediated by nicotinic acetylcholine receptors (nAChRs). Starting from the middle of the second postnatal week, retinal waves are mediated solely by GluRs (Feller et al., 1996; Bansal et al., 2000; Zhou, 2001; Demas et al., 2003; Feller and Blankenship, 2008). The spontaneous activities mediated by both nAChRs and GluRs are thought to regulate RGC dendritic development (Wong et al., 2000; Wong and Ghosh, 2002). In the retina of turtle hatchlings, chronic blockade of nAChR-mediated retinal waves with curare reduces RGC receptive fields (Sernagor and Grzywacz, 1996). In mice, genetic deletion of β2 subunits of nAChR eliminates the retinal waves mediated by nAChRs during the first postnatal week and disturbs RGC dendritic stratification during early postnatal age (Bansal et al., 2000). However, genetic deletion of ChAT eliminates the retinal waves mediated by AChRs before P5 and reduces the size of the dendritic field of ON arbor of large-field bistratified RGCs at P7–8 but does not significantly affect the OFF arbor and the size of the dendritic filed of large-field monostratified RGCs (Stacy et al., 2005).

The roles of GluR-mediated retinal waves on the development of RGC dendrites are also controversial. It has been shown that intraocular injection of APB during early postnatal ages arrests the developmental stratification and segregation of RGC dendrites in the IPL (Bodnarenko and Chalupa, 1993; Bodnarenko et al., 1995, 1999; Deplano et al., 2004). Pharmacological blockade of GluR activity by intraocular injection of AP5 and NBQX between P10–13 reduces the kinetics of RGC dendritic growth/elimination and increases the dendritic density (Xu et al., 2010). On the other hand, blockade of glutamate release from ON bipolar cells by expressing tetanus toxin (TeNT) in ON bipolar cells has no impact on the dendritic morphology of RGCs at P21, supporting the argument that glutamatergic synaptic activity does not regulate dendritic morphology of RGCs (Kerschensteiner et al., 2009). In addition, genetic deletion of the mGluR6 receptor, which blocks light evoked synaptic activity and depolarizes the membrane potential of ON bipolar cell, fails to impair dendritic stratification of mouse RGCs (Tagawa et al., 1999). There is no consistent interpretation why pharmacological blockade of GluR-mediated activity causes significant dendritic defects of RGCs while genetic inhibition of glutamate release from bipolar cells has no effect on RGC dendrites. Our current study demonstrated that cell specific mutation of NMDAR has significant effects on dendritic morphology of J-RGCs at P13, which is similar to the effects induced by intraocular injection of AP5+CNQX, but much weaker effects on the J-RGCs at P30, indicating an age-dependent mechanism.

In the CNS, synaptic activity mediated by glutamate receptors, particularly NMDARs, of newly established synapses are thought to stabilize the dendritic filopodia and promote them to grow into dendritic branches (Niell et al., 2004). Consistently, pharmacologically or genetically inactivation of NMDARs blocks the elaboration of dendrites of optic tectal neurons (Rajan and Cline, 1998), motor neurons (Inglis et al., 1998; Hebbeler et al., 2002) and barrelette cells (Datwani et al., 2002; Lee et al., 2005), reduces the dendritic pruning of dentate gyrus granule cells (Espinosa et al., 2009) and retards the segregation of whisker afferents (Datwani et al., 2002; Lee et al., 2005), and barrel cortex spiny stellate cells (Espinosa et al., 2009). NMDAR-mediated activity could regulate the dendritic development of RGCs because all RGCs express NMDARs (Guenther et al., 2004) on their dendrites (Fletcher et al., 2000; Pourcho et al., 2001; Zhang and Diamond, 2009) and activation of NMDARs regulates the light responses and AMPAR plasticity of RGCs (Zhang and Diamond, 2009; Jones et al., 2012; Stafford et al., 2014). Similar to the optic tectal neurons, barrelette cells and whisker afferents in mice (Rajan and Cline, 1998; Datwani et al., 2002; Lee et al., 2005), we found that inactivation of NMDARs blocks the developmental dendritic elongation and the expansion of the dendritic field of J-RGCs before eye opening.

The axonal filopodia is an important factor to evaluate the activity of axonal morphogenesis and synaptic formation of neurons. In zebrafish larvae, silenced axons of RGCs form more highly dynamic but short-lived filopodia in NMDAR-dependent manner (Ben Fredj et al., 2010). In the optic tectum of *Xenopus laevis*, RGCs with asynchronized stimulation have more new branches, while RGCs with synchronized stimulation have fewer new but more stable branches in a NMDAR-dependent manner (Munz et al., 2014). Similarly, blockade of the activity of glutamate receptors pharmacologically lead to a significant increase in the density of dendritic protrusions of mouse RGCs (Xu et al., 2010). J-RGCs also show a high number of dendritic protrusions when the cells are in the stage of rapid dendritic growth and dendritic field expansion before eye opening. With the stabilization of dendritic growth after eye opening, the number of dendritic protrusions decreases to a low level, indicating that, similar to axonal filopodia of RGCs, the number of dendritic protrusions is an important factor to evaluate the activity of dendritic morphogenesis. This activity-dependent change of the number of dendritic protrusions is very likely regulated by NMDARs in a cell autonomous manner on J-RGCs because mutation of NMDARs specifically on J-RGCs increases the number of dendritic protrusions during early postnatal development. Because the activity mediated by NMDARs on filopodia is thought to stabilize the newly formed filopodia and promote synaptic formation (Niell et al., 2004; Lohmann et al., 2005), it is important to further investigate whether the synaptic circuit connected to J-RGCs and the synaptic function of J-RGCs is also regulated by the NMDAR-mediated activity. Our recent experimental results provided strong evidence to support the notion that cell-specific mutation of NMDAR on J-RGCs alters the types of bipolar cell inputs to J-RGCs (Young et al., 2018).

### Visual Experience and RGC Dendritic Morphogenesis

Visual experience has been shown to regulate the development of both the dendritic architecture and synaptic function of RGCs (Tian, 2008). Light deprivation increases the density of conventional synapses in the IPL (Fisher, 1979), blocks an age-dependent remodeling of dendritic complexity of RGCs (Wingate and Thompson, 1994), retards the developmental dendritic ramification of OFF RGCs in the IPL (Tian and Copenhagen, 2003; Xu and Tian, 2007), enlarges the diameter of the dendritic field and increases the number of dendritic branches of RGCs (Sernagor and Grzywacz, 1996). Functionally, light deprivation slows down a developmental increase of spontaneous synaptic activity of RGCs (Tian and Copenhagen, 2001), retards the functional segregation of ON and OFF inputs to RGCs (Tian and Copenhagen, 2003) as well as the segregation of outputs from RGCs to dLGN neurons (Akerman et al., 2002), and reduces the size of the receptive field of RGCs (Akimov and Rentería, 2014). In addition, light deprivation decreases the levels of NR2A expression in visual cortex and the retina (Nase et al., 1999; Xue and Cooper, 2001), delays an age-dependent decline of NMDAR-mediated response of RGCs (Fox et al., 1991; Carmignoto and Vicini, 1992; Guenther et al., 2004), and prolongs NMDAR-mediated responses in the LGN of ferrets (Ramoa and Prusky, 1997). However, it is still debatable whether visual experience is required for all RGC subtypes to form normal dendritic patterns and sensory maps. A recent report shows that the spacing and the overlap of RGC receptive fields at the time of eye opening were similar to those in adult rats, indicating visual experience has little impact on the development of RGC sensory maps (Anishchenko et al., 2010).

Our results show that dark rearing retards the developmental consolidation of the size of the dendritic field of J-RGCs after eye opening, which is similar to that of the RGCs of turtle retina (Sernagor and Grzywacz, 1996) but opposite to the measurements of the size of the receptive fields of RGCs of mouse retina (Akimov and Rentería, 2014). In addition, J-RGCs of dark reared mice have increased number of dendritic protrusions. Because light deprivation reduces the strength of glutamatergic synaptic activity of RGCs (Tian and Copenhagen, 2001) and pharmacologically blockade of glutamate receptor activity increases the density of dendritic protrusions of mouse RGCs (Xu et al., 2010), the increase of the number of dendritic protrusions on J-RGCs of dark reared mice is likely the results of reduced glutamatergic synaptic activity of J-RGCs. In contrast to previous reports that dark rearing retards the developmental dendritic stratification of OFF RGCs in the outer portion of the IPL (Tian and Copenhagen, 2003; Xu and Tian, 2007), J-RGCs from dark reared mice seem to have narrowly stratified dendrites in the outer IPL. Furthermore, light deprivation has no significant effect on the dendritic length and the number of dendritic branches of J-RGCs, which are also opposite to the effects induced by light deprivation on the RGCs of turtles and hamsters (Wingate and Thompson, 1994; Sernagor and Grzywacz, 1996). All together, these results suggest that visual experience and/or GluR-mediated synaptic activity could be required for the development of the dendritic architecture and sensory maps of RGCs in a subtype specific manner, which is similar to the effects of genetic deletion of ChAT on the dendritic arbors of large-field bistratified and large-field monostratified RGCs (Stacy et al., 2005).

Although both visual experience and NMDAR-mediated activity are required for the dendritic development of RGCs and the strength of glutamatergic synaptic activity of RGCs is regulated by visual stimulation, it is unclear whether visual experience regulates the dendritic development of J-RGCs through NMDAR-mediated activity. In consistent with the idea that visual stimulation promotes the consolidation of the dendritic field of J-RGCs through NMDARs after eye opening, J-RGCs with NMDAR mutation have an increased dendritic field size similar to that of NMDAR-expressing J-RGCs raised in constant darkness while dark rearing of mice with J-RGC specific mutation of NMDARs has no additional effect on the size of dendritic field. In addition, light deprivation significantly increased the number of dendritic protrusions of NMDAR-expressing J-RGCs but not J-RGCs with the NMDAR mutation, further supporting the possibility that light stimulation might regulate the dendritic morphogenesis of J-RGCs through NMDARs as that of RGC axonal morphogenesis (Ben Fredj et al., 2010; Munz et al., 2014). Regardless what are the underlying mechanisms, defects of dendritic structure indicate potential impairments in synaptic connected of J-RGCs. Therefore, the significance of the structural defects of J-RGC dendrites to the physiology of these cells needs to be further investigated.

## Author Contributions

EE and NY: data collection, data analysis. PW: animal preparation, resource management. NT: experimental design, data analysis, manuscript preparation, research fund management.

## Conflict of Interest Statement

The authors declare that the research was conducted in the absence of any commercial or financial relationships that could be construed as a potential conflict of interest.

## Supplementary Material

FIGURE S1 The size of the dendritic field of J-RGCs increases with eccentricity. The dendritic field size of each J-RGC was quantified and correlated to the distance of the soma to the optic nerve head. **(A)** A representative image of a maximum projection of confocal image of a flat-mounted whole retina of a P60 JamB-CreER:Thy1-YFP mouse. The circles of white dotted line indicate distance of eccentricity of 500 μm, 1000 μm and 1500 μm from the optic nerve head. **(B)** The scatter plot of the DF size of J-RGCs as a function of the distance of eccentricity of 80 J-RGCs (2 mice, 3 retinas). **(C)** The average DF size of J-RGCs grouped based on their eccentricity distance. **P* < 0.05; ***P* < 0.01; and ****P* < 0.001.
