# Supplementary figures and images for: Glutamate Activity Regulates and Dendritic Development of J-RGCs
Source: Front Cell Neurosci. 2018 Aug 14;12:249. doi: 10.3389/fncel.2018.00249 (PMC6102418; doi:10.3389/fncel.2018.00249)

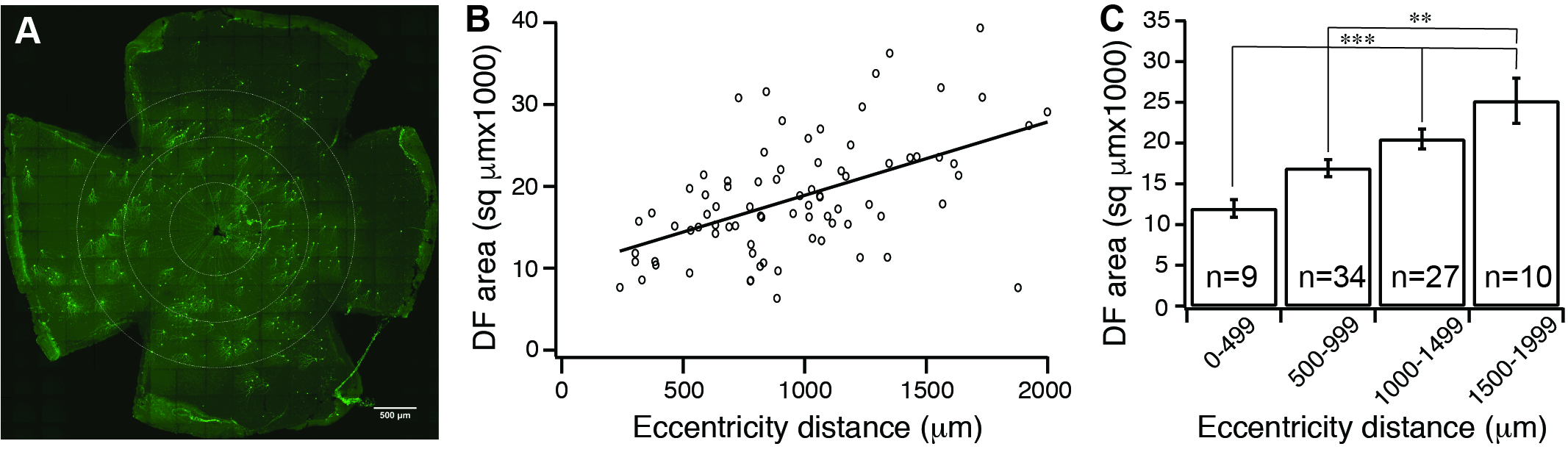

Supplement: FIGURE S1 — The size of the dendritic field of J-RGCs increases with eccentricity. The dendritic field size of each J-RGC was quantified and correlated to the distance of the soma to the optic nerve head. (A) A representative image of a maximum projection of confocal image of a flat-mounted whole retina of a P60 JamB-CreER:Thy1-YFP mouse. The circles of white dotted line indicate distance of eccentricity of 500 μm, 1000 μm and 1500 μm from the optic nerve head. (B) The scatter plot of the DF size of J-RGCs as a function of the distance of eccentricity of 80 J-RGCs (2 mice, 3 retinas). (C) The average DF size of J-RGCs grouped based on their eccentricity distance. *P < 0.05; **P < 0.01; and ***P < 0.001. [file Image_1.TIF]
